# C-terminal fragment of agrin (CAF) levels predict acute kidney injury after acute myocardial infarction

**DOI:** 10.1186/s12882-017-0611-9

**Published:** 2017-06-24

**Authors:** Spyridon Arampatzis, Georgios Chalikias, Vasilios Devetzis, Stavros Konstantinides, Uyen Huynh-Do, Dimitrios Tziakas

**Affiliations:** 1 Department of Nephrology, Hypertension and Clinical Pharmacology, Inselspital, University Hospital Bern, 3010 Bern, Switzerland; 2 Cardiology Department, Medical School, Democritus University of Thrace, Alexandroupolis, Greece

**Keywords:** Acute kidney injury, Acute myocardial infarction, C-terminal agrin fragment, Biomarkers

## Abstract

**Background:**

Patients with acute myocardial infarction are at high risk for acute kidney injury. Novel biomarkers that can predict acute kidney injury in AMI may allow timely interventions. C-terminal fragment of agrin (CAF), a proteoglycan of the glomerular and tubular basement membrane, have been recently associated with rapid renal function deterioration and proximal tubular dysfunction. It is unknown whether elevated CAF levels may serve as a novel AKI biomarker in patients presenting with AMI.

**Methods:**

In 436 persons enrolled in a multicenter prospective observational cohort study of patients with acute myocardial infarction, we measured plasma and urine levels of several kidney injury biomarkers including CAF, neutrophil gelatinase-associated lipocalin (NGAL), interleukin-18 (IL-18) and cystatin-C.The relationship between biomarker levels at baseline and the development of AKI and long-term mortality were analyzed after adjustment for demographic and clinical variables.

**Results:**

AKI incidence was up to 15% during hospitalization. The predictive accuracy for AKI of urinary CAF was similar to NGAL and superior to other tested kidney injury biomarkers. In a multivariate model that included all possible confounding variables only urinary CAF continued to be an independent marker for AKI (OR 1.35 95%CI 1.05 -1.74). During the 2 years follow-up, only plasma CAF levels remained a significant independent predictor of mortality (OR 2.5 95%CI 1.02-6.2; *P* = 0.04).

**Conclusions:**

Elevated CAF levels are associated with AKI in patients with acute myocardial infarction. Our study provides preliminary evidence that CAF levels may predict AKI and mortality after AMI in low risk patients with relative preserved kidney function at baseline.

**Electronic supplementary material:**

The online version of this article (doi:10.1186/s12882-017-0611-9) contains supplementary material, which is available to authorized users.

## Background

Acute kidney injury (AKI) is a common complication after acute myocardial infarction (AMI). Early diagnosis and subsequent management of AKI after AMI remains challenging, since the AKI etiology is multifactorial. In AMI, impaired renal function may result from deteriorating heart function, nephrotoxic contrast agents or due to underlying kidney disease [1–3]. In addition, AKI classification is primarily based on serum creatinine changes mostly occurring days after the initial insult.

The need for better biomarkers of AKI prediction has been acknowledged as a crucial barrier to improvement of the outcomes after AMI. The ideal, “troponin-like” renal injury biomarker should be able to help stratify patients at risk for AKI before any critical intervention or contrast media admission. Several urinary and plasma proteins have been proposed as potential biomarkers for predicting AKI in AMI.These include renal function markers such as cystatin C (Cyst-C) and specific renal injury markers, such as interleukin 18 (IL-18), neutrophil gelatinase-associated lipocalin (NGAL) and kidney injury molecule-1 (KIM-1) [4–7]. All these biomarkers however, are still not widely applied and have been associated with various clinical limitations particularly in low risk patients [8, 9].

Agrin which is a large proteoglycan serves as a major heparan sulfate proteoglycan for the glomerular and the tubular basement membrane and is highly expressed in the kidney. Agrin serves also as a ubiquitous component of the extracellular matrix [10]. Cleavage of agrin by the serine protease neurotrypsin at the beta site produces the 22 kDa C-terminal fragment (CAF22) [11]. Elevated serum CAF22 (sCAF) has recently emerged as a promising biomarker for kidney function and is highly associated with other renal markers in septic patients, renal transplant recipients and patients with chronic kidney disease. [11–13]. In previous studies we were able to show that baseline urinary CAF22 levels were associated with subsequent eGFR loss and proteinuria progression in diabetic nephropathy [14]. In animal models we were able to show that CAF22 was cleared from circulation primary by glomerular filtration and that filtered CAF22 was reabsorbed by the proximal tubule [15]. Based on our findings we postulated that CAF22 may represent a novel renal injury biomarker which may reflect both renal function and indirectly proximal tubular integrity.

Therefore, we performed a post-hoc analysis of a large prospective multicenter study with AMI patients in order to explore the association of CAF as early biomarkers for AKI prediction at hospital admission and to compare the diagnostic performance of CAF to established renal injury biomarkers.

## Methods

### Study design

The study design of this prospective observational cohort study has been previously published [16]. In short consecutive patients with acute ST-elevation MI (STEMI) or non ST-elevation MI (NSTEMI) were recruited if they fulfilled the following inclusion criteria: 1) age ≥ 18 years; 2) ability to provide written, informed consent; and 3) acute, spontaneous (type 1) AMI. The main exclusion criteria were presence of pre-existing renal disease and AMI-related symptom onset ≥72 h from hospital admission. Patients referred for urgent coronary artery bypass grafting and those suffering a fatal event during the index hospitalization were also excluded from the study.

Consecutive patients admitted to the Coronary Care Unit from 3 different Cardiology Departments (Alexandroupolis; Kavala; Athens) in Greece were recruited from July 2010 to May 2014 [16]. In 436 patients, baseline plasma and urine samples were available for additional assessment of CAF concentrations and 403 patients had a valid result for all plasma and urine biomarkers. STEMI patients underwent either primary percutaneous coronary intervention (PCI), or fibrinolysis followed by rescue or elective PCI as indicated [17, 18]. NSTEMI patients underwent invasive or primarily conservative (followed by elective PCI) strategy according to current recommendations [18, 19]. Patients were assessed for presence of AKI at 48 h post admission using the Acute Kidney Injury Network (AKIN) [20] and the Acute Dialysis Quality Initiative [Risk, Injury and Failure (RIFLE)] criteria [21] and also at hospital discharge using the RIFLE criteria and the Kidney Disease: Improving Global Outcomes (KDIGO) criteria [22]. Changes in absolute or relative changes in creatinine, or eGFR were presumed to have occurred within hospitalization. In addition, in each patient a transthoracic echocardiography study was performed during hospitalization using standard techniques [23]. Death from any or cardiovascular causes, repeat hospitalization and need for dialysis or other renal replacement therapy was assessed with telephone contacts during the 2-years follow up.

### Sample collection and measurement

Blood and urine sampling for CAF, FE-Na, NGAL, IL-18 and cyst-C assessment was performed at hospital admission and before any critical intervention according to previously established analytical protocols. Peripheral blood samples for measurement of blood chemistry (renal function) and full blood count were obtained from all patients on admission, 48 h after the index event, and also daily until discharge. Blood samples were drawn from a peripheral vein of the patients in vacutainer tubes containing EDTA as an anticoagulant. Samples were immediately centrifuged at 4000 rpm for 10 min at ambient temperature, and the extracted plasma was stored in aliquots and frozen at −70 °C until use. Urine samples were collected in aliquots from spot urine during admission and were immediately frozen at -70 °C until use. [24]. The coefficients of variation for intra- and inter-assay precision were <8% and <10% respectively, for all assays. CAF levels were measured using the NTtotalCAF ELISA kit from Neurotune [14]. The lower detection limit was 40 pM. Data for plasma were valid when the coefficient of variation (%CV) was below 20%. Independent analysis revealed a mixed intra- and inter-assay %CV below 12.6% for plasma samples.

### Study oversight

The study was approved by the institutional Ethics Committee, and all subjects gave written informed consent at the time of enrollment. The CAF sample kits measurements were performed blindly by Neurotune AG Schweiz, which had no role in data analysis.

### Statistical analysis

Data are presented as percentages for categorical data, as means ± standard deviation (SD) for continuous variables that were normally distributed and as medians with interquartile range (IQR) for non-normally distributed data. Comparisons between categorical variables were performed by chi- square test or Fisher’s exact test when required. Differences in continuous variables between two groups were assessed using the Student’s t-test or the Mann-Whitney’s U-test as appropriate.

The diagnostic accuracy of the under investigation biomarkers was determined by calculating the area under the curve (AUC), sensitivity, specificity, and positive and negative predictive values using receiver operating characteristic (ROC) curve analysis. The association between CAF levels and incidence of AKI during hospitalization was assessed by univariable logistic regression analyses. For both assessments serum creatinine on admission were considered as baseline. Independence of the association was assessed in multivariable logistic regression analysis models arbitrarily using variables that could act as possible cofounders for prognosis (age, diabetes mellitus, presence of anemia, glomerular filtration rate on admission, albuminuria, LV EF, invasive or not treatment strategy of the index event, presence of any adverse event during the index hospitalization and infarct size based on CPK maximum levels). For all (incidence or prognosis) logistic regression analysis models, odds ratios (OR) with 95% confidence intervals were calculated.

A *p* value <0.05 was considered to indicate statistical significance; all tests were two-sided. The IBM SPSS Statistics 20.0 statistical software package (SPSS Inc., Chicago, Illinois, USA) was used for all calculations with an exception of AUC comparison and Cochran-Armitage test for trend for which MedCalc 19.2 Statistical Software (MedCalc Software, Mariakerke, Belgium) was used.

## Results

### Baseline characteristics

Baseline demographic, clinical, angiographic and laboratory characteristics of the cohort and in AKI versus non-AKI patents according to RIFLE-Criteria are listed in Table 1 **.** The majority of the patients were managed invasively during hospitalization and one fourth of the population experienced at least one in-hospital adverse event.

**Table 1:** Demographic, clinical and angiographic data at baseline and in-hospital characteristics of study cohort

Variable	Study Cohort (*n* = 403)
Age, years	62 (13)
Age > 70 years, n(%)	132 (33)
BMI, Kg/m^2^	28 (4)
BSA, m^2 a^	1.97 (0.2)
Male/Female, n(%)	314 (78) / 89 (22)
Risk Factors	
Hypertension, n(%)	226 (56)
Diabetes mellitus, n(%)	104 (26)
Dyslipidemia, n(%)	161 (40)
Current smokers, n(%)	219 (54)
Co-morbidities	
Previous MI, n(%)	65 (16)
Chronic heart failure, n(%)	6 (1.5)
Peripheral arterial disease, n(%)	22 (6)
Previous stroke or TIA, n(%)	31 (8)
Atrial fibrillation, n(%)	
Paroxysmal	7 (1.5)
Chronic	14 (4)
Valvular disease, n(%)	5 (1)
Previous PCI, n(%)	45(11)
Previous CABG, n(%)	10 (2.5)
Anemia, n(%)	66 (16)
Laboratory Data	
Glucose at admission, mg/dl	153 (70)
Hematocrit at admission, %	42 (5)
Hemoglobin at admission, g/dL	14.2 (1.7)
Baseline creatinine, mg/dL	0.98 (0.25)
Creatinine at 48 h, mg/dL	1.03 (0.31)
Peak Creatinine during hospitalization, mg/dL	1.12 (0.47)
Baseline eGFR, mL/min ^b^	94 (35)
eGFR at 48 h, mL/min ^b^	91 (38)
Lowest eGFR during hospitalization, mL/min ^b^	85 (33)
Baseline eGFR classification, n(%)	
>90 ml/min	199 (49)
60-90 ml/min	142 (35)
<60 ml/min	62 (16)
Ejection fraction during hospitalization, n(%)	
Normal (>55%)	230 (57)
Mildly reduced (45-55%)	106 (26)
Moderately reduced (35-44%)	60 (14.5)
Severely reduced (<35%)	7 (1.5)
Total Cholesterol, mg/dL	204 (49)
LDL Cholesterol, mg/dL	129 (43)
HDL Cholesterol, mg/dL	44 (18)
Triglycerides, mg/dL	155 (98)
CPK at admission, IU/L	201 (106-566)
CK-MB at admission, IU/L	32 (19-62)
Peak CPK during hospitalization, IU/L	1130 (400-2001)
Peak CK-MB during hospitalization, IU/L	91 (37-179)
Peak CRP during hospitalization, mg/dL	4.3 (1.45-8.90)
Hospitalization Data	
Type of Acute Coronary Syndrome	
STEMI, n(%)	288 (71)
NSTEMI, n(%)	115 (29)
Site of MI	
Inferior, n(%)	129 (32)
Anterior, n(%)	169 (41.5)
Lateral, n(%)	42 (10)
Infero-lateral, n(%)	13 (3.5)
Antero-lateral, n(%)	47(11.5)
Posterior, n(%)	3 (1.5)
Heart rate at admission, bpm	80 (18)
Systolic BP at admission, mmHg	135 (26)
Diastolic BP at admission, mmHg	78 (12)
Low BP (<90 mmHg) at admission, n(%)	20 (5)
Time from symptom onset, hours	6.5 (2-11)
TIMI risk score, n	3 (1-4)
Killip class, n(%)	
Class I	368 (91)
Class II	24 (6)
Class III	8 (2)
Class IV	3 (1)
Coronary artery disease, n(%)	
Non-significant disease	17 (4)
1-vessel	218 (54)
2-vessel	103 (25)
3-vessel	65 (17)
Left main stem disease	25 (6)
ACS treatment strategy, n(%)	
Invasive during hospitalization	261 (65)
Primarily conservative	142 (35)
IV treatment with, n(%)	
Thrombolysis	172 (42)
GP IIb/IIIa inhibitors	50 (12.5)
β-blockers	38 (9.5)
Use of inotropic support (catecholamine use or balloon counterpulsation)	17 (4)
Diuretics	27 (6)
Anti-arrhythmics	48 (11.5)
Adverse events, n(%)	96 (23)
Recurrent ischemia	20 (5)
Re-infarction	14 (3.5)
Hemodynamic collapse [9]	16 (4)
Tachyarrhythmia requiring intervention	38 (9.5)
Bradyarrhythmia requiring intervention	13 (3.5)
Acute heart failure during hospitalization	31 (7)
Major bleeding complications [10]	3 (1)
Acute mitral valve regurgitation (severe)	4 (1)
Pericarditis	3 (1)
Hospitalization (days)	5 (4-7)
Prior Medication Use	
ACE- inhibitors, n(%)	128 (31)
Angiotensin receptor blockers, n(%)	89 (22)
Diuretics, n(%)	65 (16)
Aldosterone antagonists, n(%)	18 (4)
Nitrates, n(%)	36 (8)
Digitalis, n(%)	11 (2.5)
β- blockers, n(%)	123 (30.5)
Calcium channel blockers, n(%)	43 (10)
Amiodarone, n(%)	15 (3.5)
Statins, n(%)	164 (40)
Fibrates, n(%)	1 (0.2)
Aspirin, n(%)	115 (28)
P2Y12 antiplatelets, n(%)	108 (26)
Anticoagulants, n(%)	9 (2)
Anti-diabetics, n(%)	55 (13)
Metformin, n(%)	35 (8)
Insulin, n(%)	16 (4)

Values are expressed as means (with the corresponding standard deviation) or medians (with the corresponding interquartitile range) for continuous variables, and as numbers of patients and percentages for categorical variables

*ACE* angiotensin converting enzyme, *BMI* body mass index, *BP* blood pressure, *CABG* coronary artery bypass graft surgery, *CK-MB* creatinine kinase myocardial fraction, *CPK* creatine phosphokinase, *CRP* C-reactive protein, *eGFR* estimated glomerular filtration rate, *GP* glycoprotein, *HDL* high density lipoprotein, *IV* intravenous, *MI* myocardial infarction, *NSTEMI* non ST elevation myocardial infarction, *LDL* low density lipoprotein, *CAF* C- terminal agrin fragment levels, *Cyst-C* cystatin-C, *IL-18* interleukin-18, *NGAL* neutrophil gelatinase-associated lipocalin, PCI, percutaneous coronary intervention, *STEMI* ST elevation myocardial infarction, *TIA* transient ischemic attack, *TIMI* Thrombolysis in myocardial infarction

^a^Calculated using the Mosteller formula

^b^Calculated using the Cockcroft-Gault formula

### Incidence of AKI

The incidence of AKI in our study population ranged from 7% to 15% (Additional file 1: Table S1) depending on timing (at 48 h vs. during hospitalization) and on definition used (AKIN vs. RIFLE vs. KDIGO). The majority of the patients had stage 1 kidney injury whereas none of the patients required dialysis during hospitalization. For further analysis, patients were considered to have AKI using the KDIGO or RIFLE criteria during hospitalization.

### Relationship between plasma and urine concentrations of biomarkers with plasma creatinine levels and AKI

CAF concentrations in both mediums were significantly correlated with creatinine levels on admission (urine; Spearman’s rho 0.233, *P* < 0.001, plasma; Spearman’s rho 0.175, *P* < 0.001). This significant association was also observed with creatinine levels 48 h post admission (urine; Spearman’s rho 0.263, *P* < 0.001, plasma; Spearman’s rho 0.226, *P* < 0.001) and with peak creatinine levels (urine; Spearman’s rho 0.317, *P* < 0.001, plasma Spearman’s rho 0.225, *P* < 0.001) during hospitalization. The observed incidence of AKI increased across quartiles of urine CAF (Cochrane-Armitage test for trend with increasing quartile values; chi-square for trend, 10.99; *P* < 0.001). The association between increasing levels of urine CAF and the predicted incidence of AKI suggested a linear effect (r2 = 0.983). Finally, CAF concentrations were also significantly associated with Cyst-C levels both in plasma (Spearman’s rho 0.292, *P* < 0.001) and in urine (Spearman’s rho 0.267, *P* < 0.001).

### Diagnostic performance

**Fig. 1:**
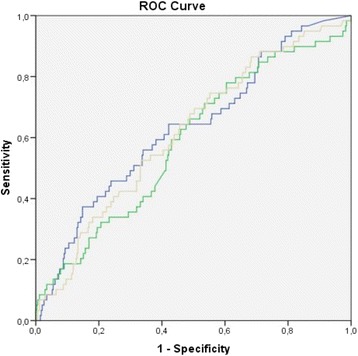
Comparison of predictive accuracy for AKI of under investigation markers using ROC analysis in the study cohort. Blue line, urinary CAF; Green line, plasma CAF; Grey line, NGAL. AKI, acute kidney injury; NGAL, neutrophil gelatinase-associated lipocalin; plasma CAF, plasma C-terminal agrin fragment

From the under investigation variables only urine NGAL, urine (u) and plasma (p) CAF were capable of detecting AKI. The discriminating ability (regarding the incidence of AKI during hospitalization) of the urine concentration of the parameters under investigation ranged from good to moderate whereas from plasma levels only plasma CAF had a moderate discriminating ability for AKI occurrence (Table 2). In comparison, urinary CAF had a similar diagnostic accuracy for AKI as assessed with ROC analysis compared to urine NGAL (*p* = 0.73), and plasma CAF (*p* = 0.38) (Fig. 1). Indexing these markers to BMI, BSA, and urine creatinine or using their urine to plasma ratio did not improve their diagnostic accuracy (Additional file 1: Table S2).

**Table 2:** Discriminating ability (area under the curve) of the under investigation variables regarding the incidence of AKI during hospitalization

	AUC	95% CI	*P* value	Sens	Spec	+PV	-PV
Urine							
IL-18	0.538	0.450-0.626	0.35				
NGAL	0.616	0.540-0.692	0.004	75	45	19	91
Cyst-C	0.573	0.489-0.657	0.07				
CAF	0.630	0.552-0.708	0.001	37	85	30	89
Plasma							
IL-18	0.530	0.440-0.619	0.47				
NGAL	0.522	0.438-0.606	0.6				
Cyst-C	0.571	0.492-0.650	0.08				
CAF	0.587	0.509-0.666	0.03	71	47	19	90

*AKI* acute kidney injury, *AUC* area under the curve, *CAF* C-terminal agrin fragment, *CI* confidence interval, *Cyst-C* cystatin-C, *IL-18* interleukin-18, *NGAL* neutrophil gelatinase-associated lipocalin, *n/a* non-applicable, *Sens* sensitivity, *Spec* specificity, *+PV* positive predictive value, *−PV* negative predictive value

### Diagnostic accuracy

Concerning diagnostic accuracy, ROC analysis identified a value of 1033 pM as optimal in predicting development of AKI. The sensitivity of urinary CAF was 37% (95%CI 25-51%) and the specificity 85% (95%CI 81-89) with a negative predictive value of 89% (95%CI 85-92%) and a positive predictive value of 30% (95%CI 20-42%). Moreover, the urinary CAF cut-off was associated with a positive likelihood ratio (+LR) of 2.52 (95% CI 1.7 -3.8) and a negative ratio (−LR) of 0.7 (95% CI,0.6 -0.9). Applying Bayes’ theorem, if we consider 15% as the pre-test probability for developing AKI, the post-test probability for developing AKI, when urinary CAF levels are ≥1033 pM, is doubled to 30% (95% CI, 22-40). Similarly, the post-test probability for developing AKI, when the urinary CAF concentrations are <1033 pM, is only 11% (95% CI, 9-13). Applying the Bayes theorem in terms of number needed to diagnose using the cut-off value of 1033 pM, 1 in 3 positive tests are truly predictive of the disease whilst 1 in 1 negative tests are truly non-predictive of the disease.

### Multivariate modeling

Levels of urinary CAF were associated with AKI incidence in a univariate model (OR per 1 SD, 1.45 95%CI 1.15-1.82, *P* = 0.002). The observed association was more robust compared to those observed with plasma CAF levels (OR per 1 SD, 1.41 95%CI 1.11-1.79, *P* = 0.005) and urine NGAL levels (OR per 1 SD, 1.15 95%CI 0.93-1.42, *P* = 0.2).

Levels of urinary CAF continued to be associated with AKI incidence in a multivariate model (Table 3) that included multiple variables (age, diabetes mellitus, presence of anemia, glomerular filtration rate on admission, albuminuria, LV EF, invasive or not treatment strategy of the index event, presence of any adverse event during the index hospitalization and infarct size based on CPK maximum levels) with an OR 1.35 95%CI 1.05 -1.74. Of interest, neither urine NGAL (*P* = 0.62), nor plasma CAF (*P* = 0.24) concentrations were independent predictors of AKI in multivariable models. The addition of urinary CAF levels in predictive models including each one of the 3 comparator biomarkers resulted in increased discriminating ability. Furthermore, the addition of urinary CAF levels to a predictive model including all 3 biomarkers (urine NGAL and plasma CAF) showed a better predictive performance. However, the additive value of urinary CAF levels was marginally non-significant. (Additional file 1: Table S3)

**Table 3:** Multivariate analysis

	Multivariate analysis		
	OR	95%CI	*P* value
Age	1.03	0.99-1.07	0.165
Diabetes Mellitus	1.83	0.96-3.51	0.067
CPK peak levels (IU/L)	1.01	0.99-1.02	0.056
Hemoglobin (g/dL)	0.81	0.65-0.99	0.003
GFR baseline (ml/min)	1.01	0.99-1.02	0.194
Albuminuria			0.295
0-30 mg/g			n/a
30-300 mg/g	1.43	0.73-2.81	0.302
> 300 mg/g	2.09	0.79-5.46	0.133
LV Ejection fraction			0.002
Normal (>55%)			n/a
Mildly reduced (45-55%)	2.01	0.98-4.12	0.057
Moderately reduced (35-44%)	3.64	1.71-9.77	0.001
Severely reduced (<35%)	7.58	1.45-39.29	0.016
Invasive vs conservative treatment	0.88	0.46-1.68	0.691
Presence of adverse event	1.56	0.78-3.12	0.208

Presence of adverse events refer to recurrent ischemia, re-infarction, hemodynamic collapse, tachyarrhythmia requiring intervention, bradyarrhythmia requiring intervention, acute heart failure during hospitalization, major bleeding complications, acute mitral valve regurgitation (severe), pericarditis

The method use was backward deletion method using the Likelihood Ratio criterion according which all variables were entered in the model and for each step the worse performing variable (according to the criterion used) was discarded. Therefore, the significant variables were, except from CAF22, hemoglobin levels, LV ejection fraction, and marginally diabetes mellitus and myocardial infract size (using CPK peak levels)

### Long-term outcome

During the 2-year follow up, 33 deaths were observed among the whole study population. Twenty-six deaths were attributed to cardiovascular causes whereas the rest 7 were attributed to other causes mainly cancer. 57 patients required at least one repeat hospitalization during the follow up period, whilst 15 patients developed deterioration in their kidney function that required an inpatient intervention or outpatient follow up. Of interest only 2 out of the 15 patients required permanent renal replacement therapy.

Only plasma CAF levels were associated with death from any cause (OR 4.1 95%CI 1.7-9.7; *P* = 0.001) whereas urinary CAF concentration were not associated (*P* = 0.84). Multivariable analysis showed that plasma CAF levels remained a significant independent predictor of mortality (OR 2.5 95%CI 1.02-6.2; *P* = 0.04). Neither plasma CAF (*P* = 0.08) nor urinary CAF (*P* = 0.8) were predictive of deterioration of kidney function albeit plasma CAF levels were marginally non-significant.

## Discussion

In this study, we analyzed and compared the precision and discriminative ability of CAF to previous reported set of biomarkers concerning AKI prediction after AMI. Our results indicate 1) that urinary CAF has an equivalent ability compared to NGAL for AKI prediction in low risk patients and 2) in multivariable analysis plasma CAF levels remained a significant independent predictor of mortality. Moreover these findings were observed in patients with relative preserved kidney function at baseline, and before any AMI related critical interventions.The association between CAF and traditional renal function and injury parameters was recently demonstrated by our group in type II diabetics and previously in septic and transplanted patients. [13, 14, 25] In this cohort of patients with relative preserved renal function CAF concentrations in urine and plasma were significantly correlated with established renal function parameters such as creatinine and Cyst-C levels on admission.

AKI is a well-recognized complication in patients with AMI and in our study population AKI occurred in up to 15%. This is in accordance to previous studies reporting a high incidence of AKI ranging from 10 to 27% based on the definition applied [26–28]. Findings from the present study showed that from the under investigation biomarkers only urine NGAL, urinary CAF and plasma CAF levels were capable of detecting AKI. The urinary CAF concentrations were characterized by a fair discriminating ability. Urine NGAL and plasma CAF were also capable of detecting AKI however with lesser potency. Although the urinary CAF AUC of 0.630 is suboptimal for short term diagnostic decisions, one should take into account the low risk profile of our cohort patients. Also in the subgroup of elderly patient (>70 years old) and those with GFR ≤ 90 ml/min, CAF provided better prognostic performance with an AUC of 0.7.

Furthermore different definitions of AKI provided a similarly robust predictive ability. Levels of urinary CAF continued to be associated with AKI incidence in a multivariate and neither urine NGAL, nor plasma CAF concentrations were independent predictors of AKI in multivariate models.

Concerning long-term outcome, patients with baseline elevated levels of plasma CAF were less likely to survive long-term. Numerous studies and meta-analysis evaluated the diagnostic significance of IL-18, NGAL and Cyst-C mainly in the context of AKI and long-term mortality after cardiac surgery and in intensive care patients with multiple comorbidities [29–31]. It is possible that the insufficient discriminatory ability of other biomarkers in the present study is attributable to the clinical characteristic of our low risk AMI population.

Although the exact mechanism of CAF production and trafficking in the kidney is currently under investigation, previous findings from animal experiments support the hypothesis that CAF may reflect both structural and functional alterations [14, 15]. After more than a decade of research in the field of “novel” biomarkers for AKI we are still in search for a troponin-like biomarker which is easily measured, independent of other biological variables, and that provides both early detection and risk stratification. A holistic approach, beyond biochemical biomarkers, that will enhance the clinical value added by patient history and risk profile is necessary to objectively individualize care and identify patients for therapeutic trials. Our previous work on the development of an easily applicable risk score model for the prediction of contrast-induced AKI and the current finding arising from this study, represent a solid basis for future clinical interventional trials [32].

Our findings have limitation since that in this observational prospective study most of the patients had stage 1 kidney injury whereas none of the patients developed AKI requiring dialysis during hospitalization. This post-hoc analysis lacks information contrast media type and volume used during the interventions. Strengths include the more liberal definition of AKI (KDIGO or RIFLE criteria met during hospitalization) allowing us to include any possible confounding factors for the development of AKI and the thorough characterization of risk factors, comorbidities and hemodynamic parameters.

## Conclusion

In conclusion, our study provides preliminary evidence that CAF levels can predict AKI and mortality after AMI in low risk patients with relative preserved kidney function at baseline.

## Additional file

Additional file 1: Table S1. Acute kidney injury incidence based on AKIN/RIFLE Criteria at 48 h after admission and based on KDIGO/RIFLE Criteria during hospitalization. **Table S2.** Diagnostic performance of markers after Indexing them to BMI, BSA, and urine creatinine or using their urine to plasma ratio. **Table S3.** Performance measures of biomarker multivariate models predicting AKI. (DOCX 16 kb)

## Abbreviations

AKI: Acute kidney injury
AMI: Acute myocardial infarction
CAF: c-terminal agrin fragment
Cyst-C: Cystatin C
IL-18: Interleukin 18
KIM-1: Kidney injury molecule-1
NGAL: Neutrophil gelatinase-associated lipocalin
