# Supplementary material for: C-terminal fragment of agrin (CAF) levels predict acute kidney injury after acute myocardial infarction
Source: BMC Nephrol. 2017 Jun 24;18:202. doi: 10.1186/s12882-017-0611-9 (PMC5483277; doi:10.1186/s12882-017-0611-9)
Supplement: Additional file 1: Table S1. — Acute kidney injury incidence based on AKIN/RIFLE Criteria at 48 h after admission and based on KDIGO/RIFLE Criteria during hospitalization. Table S2. Diagnostic performance of markers after Indexing them to BMI, BSA, and urine creatinine or using their urine to plasma ratio. Table S3. Performance measures of biomarker multivariate models predicting AKI. (DOCX 16 kb) [file 12882_2017_611_MOESM1_ESM.docx]

**Additional file 1**

**Table S1.** Acute kidney injury incidence based on AKIN/RIFLE Criteria at 48 hours after admission and KDIGO/RIFLE Criteria during hospitalization.

|  | | |
| --- | --- | --- |
|  | Incidence [n(%)] | Staging (n) |
| AKIN Criteria | 29 (7%) |  |
| Stage 1 |  | 26 |
| Stage 2 |  | 2 |
| Stage 3 |  | 1 |
| RIFLE Criteria | 27 (7%) |  |
| Stage 1 |  | 23 |
| Stage 2 |  | 3 |
| Stage 3 |  | 1 |
| Stage 4 |  | 0 |
| Using both criteria | 38 (9%) |  |
| During Hospitalization | | |
| KDIGO criteria * | 51 (13%) |  |
| Stage 1 |  | 41 |
| Stage 2 |  | 6 |
| Stage 3 |  | 4 |
| RIFLE criteria * | 47 (12%) |  |
| Stage 1 |  | 39 |
| Stage 2 |  | 6 |
| Stage 3 |  | 2 |
| Stage 4 |  | 0 |
| Using both criteria * | 59 (15%) |  |

**Table S2.** Diagnostic performance of markers after Indexing them to BMI, BSA, and urine creatinine or using their urine to plasma ratio.

|  | **AUC** | **95% CI** | **P value** |
| --- | --- | --- | --- |
| **Urine indexed to BSA** |  |  |  |
| IL-18 [pg/(mL x m^2^)] | 0.545 | 0.458-0.631 | 0.27 |
| NGAL [(ng/(mL x m^2^)] | 0.620 | 0.545-0.695 | 0.003 |
| Cyst-C [(ng/(mL x m^2^)] | 0.576 | 0.491-0.661 | 0.06 |
| CAF (pM/m^2^) | 0.633 | 0.555-0.711 | 0.001 |
| **Urine indexed to BMI** |  |  |  |
| IL-18 [(pg x m^2^)/(mL x Kg)] | 0.544 | 0.456-0.632 | 0.28 |
| NGAL [(ng x m^2^)/(mL x Kg)] | 0.619 | 0.544-0.694 | 0.003 |
| Cyst-C [(ng x m^2^)/(mL x Kg)] | 0.576 | 0.491-0.661 | 0.06 |
| CAF [(pM x m^2^)/Kg)] | 0.633 | 0.555-0.710 | 0.001 |
| **Urine indexed to urine creatinine*** | | | |
| IL-18 (pg/mg) | 0.537 | 0.478-0.595 | 0.62 |
| NGAL (ng/mg) | 0.622 | 0.569-0.675 | 0.003 |
| Cyst-C (ng/mg) | 0.572 | 0.510-0.634 | 0.08 |
| CAF [(pM x dL)/mg)] | 0.637 | 0.559 – 0.715 | 0.001 |
| **Plasma to urine ratio** |  |  |  |
| IL-18 | 0.527 | 0.442-0.611 | 0.52 |
| NGAL | 0.571 | 0.496-0.646 | 0.08 |
| Cyst-C | 0.510 | 0.426-0.594 | 0.8 |
| CAF | 0.606 | 0.531-0.680 | 0.009 |

**Table S3.** Performance measures of biomarker multivariate models predicting AKI.

|  | **-2LL** | **χ ^2^** | **df** | **c-statistic** | **P value** |
| --- | --- | --- | --- | --- | --- |
| **uNGAL (model A)** | 334.1 | 1.51 | 1 | 0.616 |  |
| **Model A + uCAF** | 326.7 | 8.94 | 2 | 0.629 | 0.74 |
| **pCAF (model B)** | 328.3 | 7.32 | 1 | 0.587 |  |
| **Model B + uCAF** | 320.1 | 15.49 | 2 | 0.633 | 0.1 |
| **uNGAL +pCAF (model C)** | 326.9 | 8.66 | 2 | 0.593 |  |
| **Model D + uCAF** | 320.1 | 15.52 | 3 | 0.634 | 0.1 |
